# Effectiveness of exercise therapy combined with traditional Chinese tuina manipulation in infants with congenital muscular torticollis: a randomized controlled trial

**DOI:** 10.3389/fped.2026.1850117

**Published:** 2026-09-14

**Authors:** Qian Zhao, Yixian Gong, Wei Zhang

**Affiliations:** 1 Department of Child Health, The Fourth Hospital of Shijiazhuang, Shijiazhuang, China; 2 Department of Child Health, Hebei Reproductive and Obstetrics Hospital, Shijiazhuang, China; 3 Department of Child Health, Shijiazhuang Maternal and Child Health Hospital, Shijiazhuang, China

**Keywords:** child health, congenital muscular torticollis, exercise therapy, infants, sternocleidomastoid muscle

## Abstract

**Objective:**

To investigate the clinical efficacy of exercise therapy combined with traditional Chinese tuina manipulation in the treatment of congenital muscular torticollis (CMT) in infants.

**Methods:**

In this randomized controlled trial, 70 infants with CMT were randomly assigned to receive either exercise therapy combined with traditional Chinese tuina manipulation (combined group, *n* = 35) or traditional Chinese tuina manipulation alone (control group, *n* = 35) for 3 months. The primary outcome was the post-treatment sternocleidomastoid muscle (SCM) thickness ratio (affected/unaffected side). Post-treatment outcomes were compared using analysis of covariance (ANCOVA), adjusting for baseline values and age at enrollment. Adjusted mean differences with 95% confidence intervals (CIs) were reported.

**Results:**

Both groups showed improvements in structural and functional outcomes after treatment. After adjustment, the combined group demonstrated a significantly lower post-treatment SCM thickness ratio than the control group (adjusted mean difference, 0.441; 95% CI, 0.287 to 0.595; *P* < 0.001). The combined group also showed a significantly smaller SCM mass diameter (adjusted mean difference, 5.02 mm; 95% CI, 4.04 to 6.01; *P* < 0.001), a smaller head tilt angle (adjusted mean difference, 4.98°; 95% CI, 1.77 to 8.19; *P* = 0.003), greater cervical rotation range of motion (adjusted mean difference, −7.41°; 95% CI, −9.21 to −5.61; *P* < 0.001), and a higher muscle function scale ratio (adjusted mean difference, 0.148; 95% CI, 0.026 to 0.270; *P* = 0.018) than the control group. The overall response rate was significantly higher in the combined group than in the control group (94.3% vs. 65.7%, *P* = 0.003).

**Conclusion:**

Exercise therapy combined with traditional Chinese tuina manipulation was associated with improvements in muscle structure and cervical function in infants with CMT, leading to enhanced clinical efficacy and demonstrating promising potential clinical utility.

## Introduction

Congenital muscular torticollis (CMT) is one of the most common musculoskeletal disorders in infants. It is primarily characterized by unilateral fibrosis, shortening, or contracture of the sternocleidomastoid muscle (SCM), which leads to ipsilateral head tilt, contralateral facial rotation, and varying degrees of limitation in cervical range of motion (1). Epidemiological studies have reported that the incidence of CMT ranges from approximately 0.3% to 2.0%. If not treated in a timely manner, it may lead to craniofacial asymmetry, postural abnormalities, and even functional impairments, thereby adversely affecting long-term growth and development in affected infants (2, 3).

At present, conservative treatment remains the first-line approach for CMT, particularly in infants younger than 3 months, in whom early intervention has been shown to yield significantly better outcomes than delayed treatment (4). Among these, physical therapy (PT) has been recommended as a first-line treatment strategy in multiple clinical guidelines, primarily including passive stretching, postural correction, and caregiver-administered home exercises (1). Previous studies have demonstrated that standardized exercise therapy can significantly improve cervical rotation and lateral flexion in affected infants, while also promoting structural recovery of the SCM, as evidenced by reductions in muscle thickness and improvements in the thickness ratio between the affected and unaffected sides (5). Although exercise therapy has achieved favorable clinical outcomes, some infants still experience prolonged recovery periods or insufficient functional improvement. Therefore, optimizing treatment strategies and further enhancing therapeutic efficacy have become important focuses of current research.

Traditional Chinese tuina manipulation, as a non-pharmacological intervention, has been widely used in the treatment of CMT in infants for many years. Its core techniques include kneading, pinching, and traction manipulations, which aim to relieve local soft tissue adhesions, improve muscle blood circulation, and promote muscle fiber elongation, which may help alleviate muscle contracture. In recent years, increasing attention has been paid to the role of traditional Chinese tuina manipulation in the management of CMT. Previous studies have indicated that tuina manipulation combined with conventional stretching yields more pronounced improvements in cervical range of motion and head posture compared with stretching alone (4).

However, clinical studies investigating the combined effects of exercise therapy and traditional Chinese manipulative therapy for CMT remain relatively limited. Most existing studies have focused on single outcome measures and lack a systematic evaluation from both structural and functional perspectives. Furthermore, there is insufficient evidence regarding whether combined interventions can produce significant short-term improvements in SCM structural changes (e.g., muscle thickness and mass size) as well as functional indicators (e.g., cervical range of motion and head tilt angle).

Based on the above, this study employed a randomized controlled design to compare the clinical efficacy of traditional Chinese manipulative therapy alone with that of exercise therapy combined with traditional Chinese manipulative therapy in infants with CMT. A systematic evaluation was conducted from both structural and functional perspectives, and the effects on overall clinical outcomes were further analyzed, aiming to provide evidence-based guidance for optimizing early intervention strategies for CMT.

## Methods

### Study design

This study was a prospective randomized controlled clinical trial, and the study protocol was approved by the Ethics Committee of The Fourth Hospital of Shijiazhuang. Written informed consent was obtained from the legal guardians of all participants after they were fully informed of the study procedures, potential benefits, and risks. Eligible infants were randomly assigned using a random number table method to either the traditional Chinese tuina manipulation group (control group, *n* = 35) or the exercise therapy combined with traditional Chinese tuina manipulation group (combined group, *n* = 35). The random allocation sequence was generated by an independent statistician. Sequentially numbered, opaque, sealed envelopes were used for group allocation. Investigators responsible for participant enrollment and baseline assessment were blinded to the allocation sequence. The overall study flowchart is shown in Figure 1.

**Figure 1:**
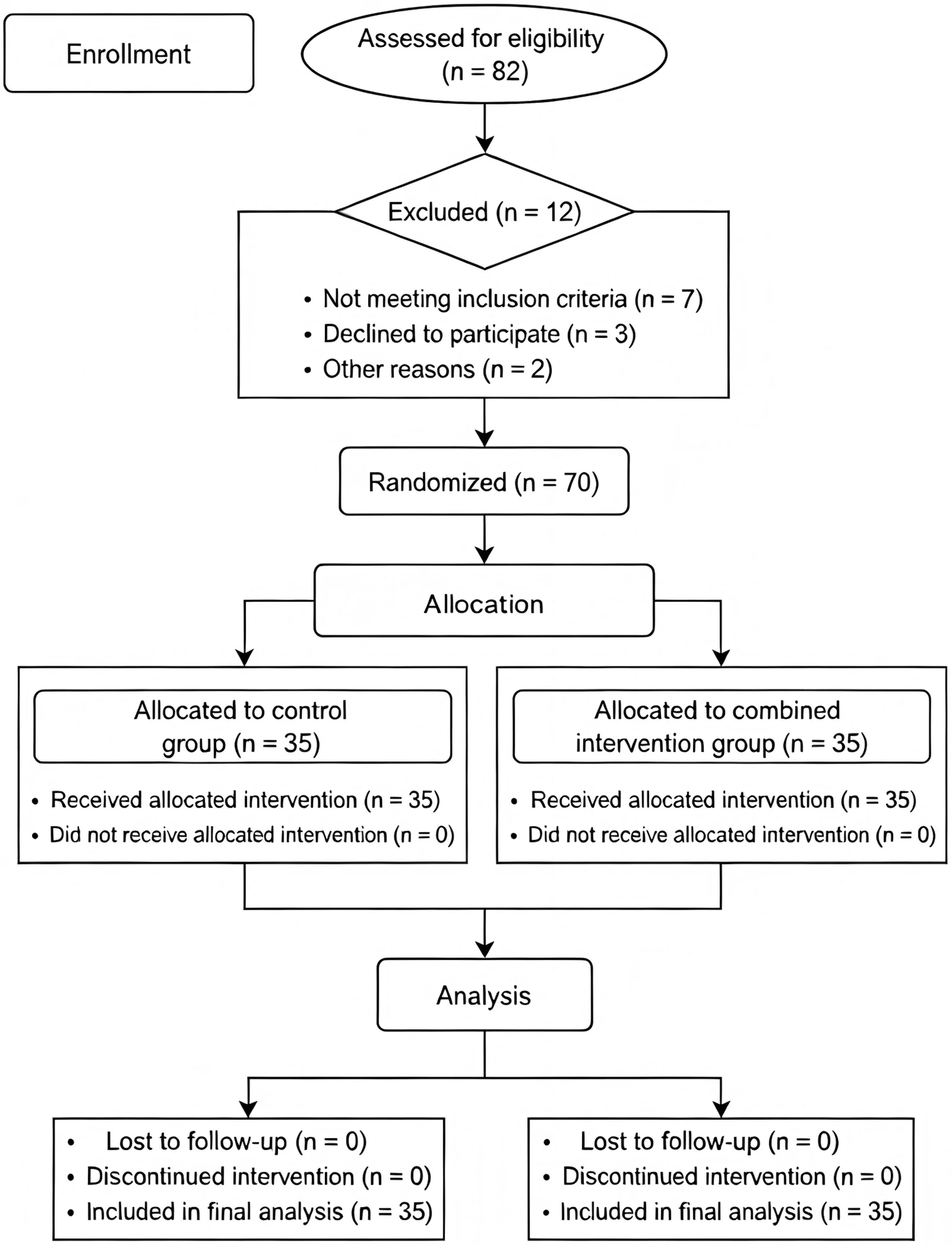
Flowchart of participant enrollment, randomization, allocation and analysis for this randomized controlled trial of infants with congenital muscular torticollis.

Outcome measures were categorized into three types: structural outcomes, functional outcomes, and clinical efficacy. Structural outcomes were assessed at baseline and after 3 months of treatment, including mass diameter, SCM thickness on the affected side, and SCM thickness ratio (affected/unaffected side). Functional outcomes were also evaluated at baseline and after 3 months, including head tilt angle, cervical rotation angle on the affected side, lateral flexion angle on the affected side, and muscle function scale (MFS) ratio (affected/unaffected side). Clinical efficacy was assessed at 3 months after treatment and was classified into three levels: cured, improved, and not improved.

### Participants

Inclusion criteria were as follows: (1) infants meeting the diagnostic criteria for congenital muscular torticollis (CMT), presenting with typical clinical manifestations such as head tilt and restricted passive cervical range of motion (lateral flexion and/or rotation); (2) age less than 1 year at enrollment; and (3) legal guardians voluntarily agreeing to participate in the study and providing written informed consent. Exclusion criteria included: (1) non-muscular forms of torticollis, such as ocular torticollis, bony torticollis, neurogenic torticollis, or habitual torticollis; and (2) infants with cervical skin lesions or concurrent infectious diseases who were unable to tolerate the treatment procedures. Sample size estimation was performed based on the sternocleidomastoid muscle (SCM) thickness ratio, which was considered the primary outcome of this study. According to previous studies on physical therapy intervention for congenital muscular torticollis, the expected between-group difference in SCM thickness ratio after treatment was estimated to be 0.15, with a standard deviation of 0.18. Using a two-sided independent samples *t*-test with a significance level of *α* = 0.05 and a statistical power of 80% (1−*β* = 0.80), the minimum required sample size was calculated to be 55 participants. Considering a potential dropout rate of 15%, a total of 70 infants were ultimately enrolled.

### Intervention

Treatments for both groups were delivered by professionally trained rehabilitation therapists and traditional Chinese medicine (TCM) practitioners, once daily, 5 days per week, for a duration of for 3 consecutive months, with continuous standardized therapy and no interruptions. Caregivers of infants in both groups received standardized home rehabilitation instructions, including positioning management, feeding posture adjustment, and caregiver-assisted passive stretching exercises. In the combined intervention group, each session began with exercise therapy, followed by a 30-minute rest, and then TCM tuina manipulation, with all procedures performed while the infants were calm and cooperative. Exercise therapy included passive cervical range of motion (ROM) training, strengthening exercises for the cervical and surrounding muscles, and symmetrical developmental movement facilitation. Passive cervical ROM training was performed with the infant in the supine position, the therapist seated at the head supporting both sides of the head, and the head slowly rotated to bring the chin toward the plane of the affected shoulder; each stretch was held for 30 s, followed by 30 s of relaxation, repeated for three sets. Strengthening exercises were conducted in the supine position, with the therapist supporting the shoulders, turning the infant 90° to the side, assisting to a seated position, and returning to supine, with ten repetitions for each side per set and three sets in total. Symmetrical developmental movement facilitation involved crossing the lower limbs while the infant was supine to elicit prone positioning and encourage head turning toward the affected side, with ten repetitions for each side per set and three sets performed. Each treatment session lasted 25 min.

TCM tuina manipulation included rubbing and pushing, pinching and grasping, traction, and rotation techniques. The total duration of TCM tuina manipulation per session was 25 min, which consisted of four standardized techniques as follows: Rubbing and pushing were applied along the origin-to-insertion path of the affected sternocleidomastoid (SCM) using the index, middle, and ring fingers at a frequency of 120 strokes per minute for 5 min. Pinching and grasping were performed with the thumb, index, and middle finger pads, repeatedly pinching and flicking the SCM at 120 strokes per minute for 5 min. Traction was applied with one hand stabilizing the affected shoulder and the other supporting the unaffected side of the head, gently pulling the head in the opposite direction for 10 s per repetition, repeated 20 times. Rotation involved caregiver-assisted shoulder stabilization while the therapist positioned one hand on the unaffected temporal region and the other supporting the affected mandible, slowly rotating the head toward the affected side within physiological tolerance, repeated 60 times. In the control group, only TCM tuina manipulation was applied, with the same techniques, duration, and frequency as in the combined group, without any exercise therapy. During the intervention period, adverse events related to exercise therapy and TCM tuina manipulation, including excessive crying, skin injury, muscle spasm, respiratory discomfort, vomiting, irritability, or treatment intolerance, were monitored and recorded throughout the study.

### Outcome measurements

Structural and functional outcomes were assessed in both groups at baseline and after 3 months of treatment, while clinical efficacy was evaluated at 3 months post-treatment. All assessments were independently performed by two professional physicians, and the mean of their measurements was used as the final result. Before the study, both evaluators received standardized training on all measurement procedures to improve measurement consistency and reduce inter-observer variability. Both evaluators were blinded to participant group allocation throughout the assessment process.

Structural outcomes were measured using color Doppler ultrasonography to determine the diameter of the SCM mass on the affected side. All ultrasound examinations were performed with the infant in a calm supine position by two trained physicians using standardized procedures. The ultrasound probe was placed longitudinally along the affected SCM, and measurements were obtained at the thickest portion of the muscle. Each measurement was repeated three times, and the mean value was used for analysis. A computerized ultrasound system was used to measure SCM thickness on both the affected and unaffected sides, from which the SCM thickness ratio (affected/unaffected side) was calculated.

Functional outcomes included head tilt angle, cervical range of motion, and muscle function scale (MFS) ratio. Head tilt angle was measured using a large goniometer as the angle between the longitudinal axis of the head and the longitudinal axis of the body in the infant's natural supine position. All angle measurements were performed while the infants were calm and positioned consistently to minimize measurement error related to posture and cooperation. Each measurement was repeated three times by two independent evaluators, and the mean value was used for analysis. Cervical range of motion was measured with the same goniometer, including rotation on the affected side and lateral flexion on the unaffected side. The muscle function scale (MFS) was a categorical ordinal scale designed to assess the functional status of infant cervical lateral flexor muscles, with scores determined by the head righting reflex (body righting reflex) to evaluate cervical lateral flexor muscle strength. For the MFS assessment, the therapist maintained the infant's trunk in an upright position and then tilted the trunk toward the affected side, observing the infant's head correction response. The scoring criteria were defined as follows: 0 points = head position below the horizontal line or failure to correct head posture in an upright position; 1 point = head aligned with the horizontal line; 2 points = head slightly above the horizontal line; 3 points = head significantly above the horizontal line; 4 points = head far above the horizontal line; 5 points = ability to correct head posture when the infant is in a horizontal position. A valid score required the infant to maintain the corresponding posture for at least 5 s; otherwise, the score was recorded as the next lower grade. Neck muscle function was scored using a cervical lateral flexor MFS for both sides, and the affected/unaffected side score ratio was calculated.

Clinical efficacy was evaluated at 3 months after treatment according to the pediatric CMT criteria described in the Shanghai Standard of Traditional Chinese Medicine Disease Diagnosis and Treatment and was used as a supplementary clinical outcome. Outcomes were classified as cured, improved, or not improved. Cured was defined as free bilateral head and neck rotation, normal range of motion, maintenance of a neutral head position for prolonged periods, and resolution of deformity. Improved was defined as near-normal cervical range of motion with the ability to maintain a neutral position, but with residual mild habitual head tilt (<10°) or an improvement of ≥15° compared with baseline. Not improved was defined as persistent inability to maintain a neutral head position or recurrence of torticollis after short-term improvement. Clinical efficacy was independently evaluated by two physicians blinded to group allocation, and the final result was determined based on consensus between the two evaluators. The total effective rate was calculated as: (Number of cured + improved cases)/(Total number of cases) × 100%.

### Statistical analysis

All statistical analyses were performed using SPSS version 26.0 (IBM Corp., Armonk, NY, USA). Continuous variables were expressed as mean ± standard deviation (SD), whereas categorical variables were presented as frequencies and percentages [n (%)]. The assumptions of normality and homogeneity of variance were assessed using the Shapiro–Wilk test and Levene's test, respectively, before conducting parametric analyses. Baseline continuous variables were compared between groups using the independent-samples *t*-test, whereas categorical variables were compared using the chi-square (*χ*^2^) test. Within-group changes in structural outcomes (SCM mass diameter, affected-side SCM thickness, and SCM thickness ratio) and functional outcomes (head tilt angle, cervical rotation range of motion, cervical lateral flexion range of motion, and MFS ratio) before and after the 3-month intervention were analyzed using the paired-samples *t*-test. The SCM thickness ratio was predefined as the primary outcome, whereas all remaining structural and functional outcomes were considered secondary outcomes. Post-treatment structural and functional outcomes were compared between groups using analysis of covariance (ANCOVA), with the post-treatment value as the dependent variable, treatment group as the fixed factor, and the corresponding baseline value together with age at enrollment as covariates. Adjusted mean differences with 95% confidence intervals (CIs) and partial eta squared (*η*p^2^) were reported to estimate treatment effects. Pearson correlation analysis was performed to evaluate the association between age at enrollment and the post-treatment SCM thickness ratio (affected/unaffected side). Clinical efficacy was compared using the chi-square (*χ*^2^) test. All statistical tests were two-sided, and a *P* value < 0.05 was considered statistically significant.

## Results

### Baseline characteristics

There were no significant differences between the two groups in general baseline characteristics, including postnatal age, sex distribution, and affected side, as well as baseline structural and functional parameters, including SCM mass diameter, affected-side SCM thickness, SCM thickness ratio, head tilt angle, cervical rotation and lateral flexion angles, and muscle function scale (MFS) ratio (all *P* > 0.05). These results indicate that the two groups were comparable at baseline (Table 1).

**Table 1:** General data and baseline indicators of two groups.

Variables	Control (*n* = 35)	Combined (*n* = 35)	*P* value
Age (days)	43.14 ± 16.00	46.11 ± 13.46	0.405
0–45 days	19 (54.3%)	16 (45.7%)	
46–90 days	16 (45.7%)	19 (54.3%)	
>90 days	0 (0.0%)	0 (0.0%)	
Male, *n* (%)	18 (51.4%)	17 (48.6%)	0.811
Left-sided involvement, *n* (%)	16 (45.7%)	18 (51.4%)	0.632
Mass diameter (mm)	21.68 ± 4.11	20.88 ± 3.97	0.836
Thickness of SCM on affected side (mm)	16.11 ± 5.87	16.05 ± 5.63	0.833
SCM thickness ratio (affected/contralateral)	2.59 ± 0.65	2.44 ± 0.62	0.827
Head tilt angle (°)	26.76 ± 13.00	27.22 ± 12.71	0.926
Cervical rotation angle on affected side (°)	48.91 ± 6.88	48.39 ± 6.36	0.667
Cervical lateral flexion angle on affected side (°)	49.52 ± 13.78	50.65 ± 15.12	0.590
Muscle function scale ratio (affected/contralateral)	2.27 ± 0.54	2.21 ± 0.51	0.867

### Comparison of structural outcomes before and after treatment

The structural outcomes before and after treatment and the within-group comparisons are presented in Table 2. After 3 months of intervention, both the control and combined groups showed significant improvements in SCM mass diameter, affected-side sternocleidomastoid muscle (SCM) thickness, and SCM thickness ratio compared with baseline (all within-group *P* < 0.05). Between-group comparisons were performed using analysis of covariance (ANCOVA), with adjustment for baseline structural parameters and age at enrollment (Table 3). For the primary outcome, the combined group showed a significantly lower SCM thickness ratio than the control group (*P* < 0.001). Among the secondary structural outcomes, SCM mass diameter was also significantly smaller in the combined group (*P* < 0.001), whereas no significant between-group difference was observed in affected-side SCM thickness (*P* = 0.069). To further evaluate the influence of age at intervention on structural recovery, Pearson correlation analysis was performed between age at enrollment and the post-treatment SCM thickness ratio (affected/unaffected side). A significant positive correlation was observed (*r* = 0.675, *P* < 0.001), indicating that infants who initiated treatment at a younger age tended to achieve greater improvement in SCM structural recovery (Figure 2).

**Table 2:** Structural outcome indicators before and after treatment in two groups.

Variables	Time	Control (*n* = 35)	Combined (*n* = 35)	*P* value
Mass diameter (mm)	Before treatment	21.68 ± 4.11	20.88 ± 3.97	0.836
	After 3 months	14.39 ± 1.29	5.21 ± 0.79	<0.001
	Within-group *P* value	<0.001	<0.001	
Thickness of SCM on affected side (mm)	Before treatment	16.11 ± 5.87	16.05 ± 5.63	0.833
	After 3 months	13.15 ± 1.68	10.84 ± 1.51	<0.001
	Within-group *P* value	0.006	<0.001	
SCM thickness ratio (affected/contralateral)	Before treatment	2.59 ± 0.65	2.44 ± 0.62	0.827
	After 3 months	1.77 ± 0.12	1.05 ± 0.66	<0.001
	Within-group *P* value	<0.001	<0.001	

**Table 3:** Adjusted intergroup comparisons of structural indicators (ANCOVA).

Variables	*P* value	Adjusted mean difference(95%CI)	*η*p^2^
Mass diameter (mm)	<0.001	5.02 (4.04,6.01)	0.496
Thickness of SCM on affected side (mm)	0.069	1.327(−0.104,2.757)	0.033
SCM thickness ratio	<0.001	0.441 (0.287,0.595)	0.158

**Figure 2:**
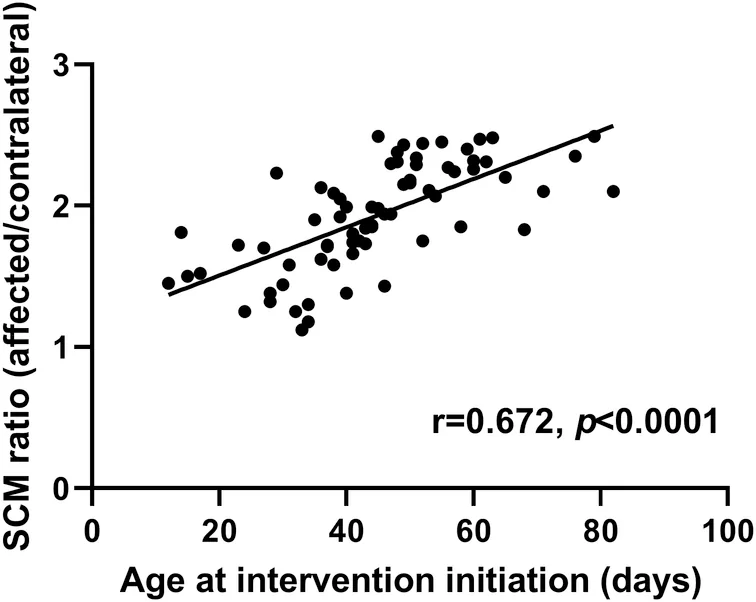
Association between age at intervention initiation (days) and post-treatment sternocleidomastoid muscle (SCM) thickness ratio (affected/contralateral side).

### Comparison of functional outcomes before and after treatment

The functional outcomes before and after treatment and the within-group comparisons are presented (Table 4, Figure 3). After 3 months of intervention, significant improvements were observed in all functional outcomes in both groups compared with baseline (all within-group *P* < 0.05). Between-group comparisons were performed using analysis of covariance (ANCOVA), with adjustment for baseline functional parameters and age at enrollment (Table 5). The adjusted analyses showed that the combined group achieved significantly better outcomes in head tilt angle, cervical rotation range of motion, and the Muscle Function Scale (MFS) ratio than the control group (all *P* < 0.05). However, no significant between-group difference was observed in cervical lateral flexion range of motion (*P* = 0.080).

**Table 4:** Functional indicators before and after treatment (within-group comparisons).

Variables	Time	Control (*n* = 35)	Combined (*n* = 35)
Head tilt angle (°)	Before treatment	26.76 ± 13.00	27.22 ± 12.71
	After 3 months	13.27 ± 4.62	3.43 ± 2.82
	Within-group *P* value	<0.001	<0.001
Cervical rotation range of motion (°)	Before treatment	48.91 ± 6.88	48.39 ± 6.36
	After 3 months	65.54 ± 5.36	81.06 ± 5.43
	Within-group *P* value	<0.001	<0.001
Cervical lateral flexion range of motion (°)	Before treatment	49.52 ± 13.78	50.65 ± 15.12
	After 3 months	57.06 ± 10.39	64.03 ± 12.39
	Within-group *P* value	0.002	0.001
Muscle function scale ratio (affected/contralateral)	Before treatment	2.27 ± 0.54	2.21 ± 0.51
	After 3 months	1.94 ± 0.22	1.72 ± 0.26
	Within-group *P* value	0.003	<0.001

**Figure 3:**
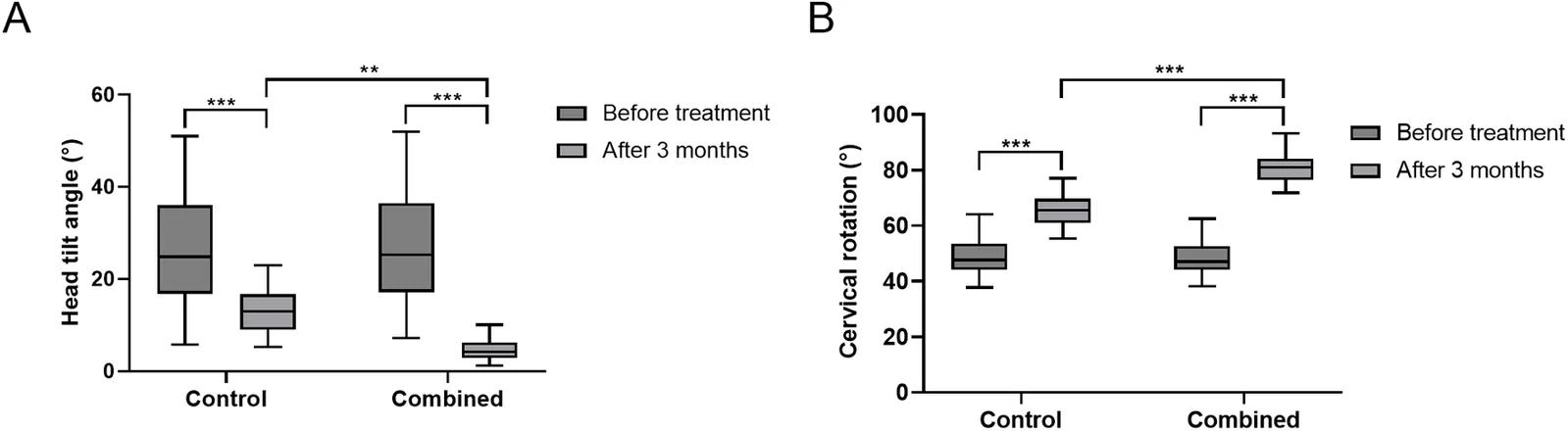
Comparisons of head tilt angle **(A)** and cervical rotation range of motion **(B)** before treatment and after 3 month intervention between the control group and combined intervention group. **p < 0.01, ***p < 0.001.

**Table 5:** Adjusted intergroup comparisons of functional indicators (ANCOVA).

Variables	*P* value	Adjusted mean difference(95%CI)	*η*p^2^
Head tilt angle (°)	0.003	4.98 (1.77,8.19)	0.221
Cervical rotation range of motion (°)	<0.001	−7.41(−9.21,−5.61)	0.432
Cervical lateral flexion range of motion (°)	0.080	−3.95(−8.38,0.48)	0.030
Muscle function scale ratio (affected/contralateral)	0.018	0.148 (0.026,0.270)	0.016

### Comparison of clinical efficacy

After 3 months of treatment, the total effective rate in the combined group was 94.3%, which was significantly higher than that in the control group (65.7%, *P* < 0.05). In terms of efficacy grading, the cure rate in the combined group was 34.3%, notably higher than 17.1% in the control group, while the not-improved rate was 5.7%, significantly lower than 34.3% in the control group. These findings suggest that the combined intervention may be associated with improved overall clinical efficacy compared with TCM manipulation alone. (Table 6). No serious adverse events related to exercise therapy or TCM tuina manipulation were observed during the intervention period. All infants completed the treatment with with acceptable tolerance.

**Table 6:** Comparison of clinical efficacy between two groups.

Group	Cured	Improved	Not improved	Overall response
Control (*n* = 35)	6 (17.1%)	17 (48.6%)	12 (34.3%)	23 (65.7%)
Combined (*n* = 35)	12 (34.3%)	21 (60.0%)	2 (5.7%)	33 (94.3%)

## Discussion

In this study, infants receiving exercise therapy combined with TCM tuina manipulation tended to show greater improvements in structural and functional outcomes, as well as overall clinical efficacy, compared with those receiving TCM manipulation alone. Specifically, the combined group exhibited greater reductions in SCM thickness ratio and mass diameter, more pronounced decreases in head tilt angle, and greater improvements in cervical rotation and muscle function. In addition, the combined group showed a higher overall response rate according to the predefined clinical efficacy criteria. These findings suggest that the combined intervention may provide additional clinical benefits compared with TCM manipulation alone.

The core pathological features of CMT are unilateral SCM fibrosis, contracture, and localized connective tissue hyperplasia, which are the primary causes of head tilt and restricted cervical mobility in affected infants (6–9). Ultrasound imaging has also confirmed characteristic changes in CMT, including SCM thickening, mass formation, and abnormal echogenicity (10–14). Consistently, baseline structural and functional abnormalities were observed in both groups in this study. The greater structural improvements observed in the combined group may be related to the synergistic effects of exercise therapy and TCM tuina manipulation. Based on the TCM principle of “promoting blood circulation, removing stasis, and relaxing muscles and tendons,” tuina manipulation may help release local soft tissue adhesions, improve microcirculation, remove inflammatory metabolites, and extend contracted fibers through stretching and rotation, which may contribute to structural recovery. Passive stretching in exercise therapy may apply mechanical tension that facilitates SCM remodeling and potentially reduces fibrotic deposition, while active strength training and symmetrical developmental exercises enhance cervical muscle balance, further optimizing structural recovery (5, 15). At the tissue and cellular levels, the mechanical tension generated by controlled passive stretching may be sensed and converted into intracellular biochemical signals through mechanotransduction. Mechanical loading may influence the behavior of fibroblasts and other resident connective-tissue cells, potentially regulating extracellular matrix turnover and promoting more organized collagen fiber alignment and remodeling. In the context of CMT, these mechanically induced remodeling processes may help reduce the persistence of disorganized fibrotic tissue and improve the mechanical compliance of the affected SCM. Meanwhile, the microcirculatory improvement and reduction in local tissue tension potentially induced by tuina manipulation may create a more favorable tissue environment for mechanically mediated remodeling. Therefore, the combination of improved local perfusion and mechanical stimulation may provide a potential biological basis for the greater structural improvements observed with the combined intervention. Together, these approaches may contribute to a sequential “release–extension–remodeling” process in which tuina manipulation may first facilitate soft-tissue relaxation and local microcirculatory improvement, passive stretching may subsequently provide controlled mechanical stimulation for tissue remodeling, and active exercise may further promote functional adaptation and muscle balance. This sequential process may help explain why the combination of manual and exercise-based interventions could produce greater structural benefits than TCM tuina manipulation alone. Meanwhile, the combined group showed greater improvements in functional outcomes, including head tilt angle, cervical range of motion, and muscle function scale ratio, which may reflect concurrent improvements in structural recovery and functional reconstruction. Functional impairment in CMT arises not only from mechanical SCM contracture but also from cervical muscle imbalance and neuromuscular control deficits. If left untreated, these deficits may impede sensorimotor integration and motor development in affected infants (16, 17). Tuina manipulation may help alleviate muscle spasm and reduce tissue tension, creating favorable conditions for functional recovery, while exercise therapy may help improve cervical joint mobility through passive ROM training, correct muscle imbalance via active strength training, and may facilitate central motor control adaptation through symmetrical developmental exercises. By simultaneously optimizing muscle mechanical properties and neuromuscular function, the combined intervention may facilitate improvements in cervical mobility in infants with CMT, consistent with previous studies reporting beneficial effects of exercise therapy (1, 5).

Most conservative treatments for CMT reported in the literature focus on single-modality interventions. Physical therapy is recommended as a first-line approach by clinical guidelines, which emphasize the importance of early standardized intervention (18). Early systematic passive stretching training can also improve SCM structure and cervical range of motion (19). While tuina combined with conventional stretching has been shown to be superior to stretching alone (4, 20), prior studies often focus on single outcome measures, lacking multidimensional evaluation of structural, functional, and clinical outcomes. In the present study, the combined exercise therapy and tuina manipulation intervention was systematically assessed across three dimensions—structure, function, and clinical efficacy—thereby supplementing existing evidence regarding combined conservative interventions for CMT. In addition, correlation analysis demonstrated that age at enrollment was significantly positively associated with the post-treatment SCM thickness ratio, suggesting that infants who initiated treatment at a younger age achieved better structural recovery. This finding is consistent with current clinical guidelines emphasizing early intervention for CMT and further supports the importance of initiating conservative treatment during the early stage of the disease. It is noteworthy that, despite the generally favorable outcomes observed with the combined intervention, individual responses may vary, influenced by factors such as the degree of muscle fibrosis, age, side of torticollis, and postural preferences. Future studies could explore individualized intervention strategies, for example, tailoring exercise intensity and frequency based on SCM thickness, mass size, and muscle strength assessment to optimize therapeutic outcomes.

Although this study was designed as a randomized controlled trial, several limitations should be acknowledged. First, this was a single-center study with a relatively small sample size, which may limit the generalizability of the findings. Second, spontaneous recovery associated with the natural growth and development of infants, together with standardized home positioning guidance provided to all caregivers, may have acted as potential confounding factors. Therefore, the within-group improvements observed in each treatment arm cannot be entirely attributed to the in-hospital interventions, and the superior outcomes in the combined therapy group should be interpreted as the additional benefit of exercise therapy when added to TCM tuina manipulation and standardized home care. Third, although SCM thickness ratio was predefined as the primary outcome and the sample size calculation was based on this endpoint, multiple secondary structural and functional outcomes were also evaluated. Therefore, the findings related to secondary outcomes should be interpreted with appropriate caution and require further confirmation in larger studies. Finally, the follow-up period was relatively short (3 months), precluding evaluation of the long-term efficacy of the interventions, the prevention of craniofacial asymmetry, and the risk of recurrence. Future multicenter studies with larger sample sizes and longer follow-up periods are warranted to further validate the present findings. In addition, incorporating objective assessments of motor function and standardized evaluation instruments would further improve the methodological rigor of clinical trials involving congenital muscular torticollis.

## Conclusion

The findings suggest that adding exercise therapy to traditional Chinese tuina manipulation may provide additional short-term benefits in structural and functional outcomes in infants with CMT. However, because the combined intervention involved greater treatment exposure and the study had limited blinding and short follow-up, further well-controlled trials are needed to confirm the specific contribution of exercise therapy.

## Data Availability

The original contributions presented in the study are included in the article/Supplementary Material, further inquiries can be directed to the corresponding author/s.

## References

[1] Kaplan SL Coulter C Sargent B. Physical therapy management of congenital muscular torticollis: a 2018 evidence-based clinical practice guideline from the APTA academy of pediatric physical therapy. Pediatr Phys Ther. (2018) 30(4):240–90. 10.1097/PEP.0000000000000544 30277962 PMC8568067

[2] Sargent B Coulter C Cannoy J Kaplan SL. Physical therapy management of congenital muscular torticollis: a 2024 evidence-based clinical practice guideline from the American physical therapy association academy of pediatric physical therapy. Pediatr Phys Ther. (2024) 36(4):370–421. 10.1097/PEP.0000000000001114 39356257

[3] Rodriguez-Huguet M Rodriguez-Almagro D Rosety-Rodriguez MA Vinolo-Gil MJ Ayala-Martinez C Gongora-Rodriguez J. Effectiveness of the treatment of physiotherapy in the congenital muscular torticollis: a systematic review. Children. (2024) 11(1):8. 10.3390/children11010008 PMC10814226 38275429

[4] Tang W Li Z Xu W Ye Y Wang H Wang Y. Effect of massage therapy on infants with congenital muscular torticollis: a retrospective comparative study. Front Pediatr. (2023) 10:984675. 10.3389/fped.2022.984675 36699315 PMC9869063

[5] Song S Hwang W Lee S. Effect of physical therapy intervention on thickness and ratio of the sternocleidomastoid muscle and head rotation angle in infants with congenital muscular torticollis: a randomized clinical trial (CONSORT). Medicine. (2021) 100(33):e26998. 10.1097/MD.0000000000026998 34414985 PMC8376309

[6] Xiong Z Zeng S Chen H Qiu X Tang G Tang Y. Unique finding in congenital muscular torticollis: clinic screening on the neck of one day old neonate and ultrasonographic imaging from birth through 3 years of follow-up. Medicine. (2019) 98(11):e14794. 10.1097/MD.0000000000014794 30882654 PMC6426618

[7] Hwang D Shin YJ Choi JY Jung SJ Yang SS. Changes in muscle stiffness in infants with congenital muscular torticollis. Diagnostics. (2019) 9(4):158. 10.3390/diagnostics9040158 31652674 PMC6963363

[8] Chen H Tang S Gao F Xu JL Jiang X Cao J. Fibrosis, adipogenesis, and muscle atrophy in congenital muscular torticollis. Medicine. (2014) 93(23):e138. 10.1097/MD.0000000000000138 25415668 PMC4616345

[9] Uluer MC Bojovic B. A rare cervical dystonia mimic in adults: congenital muscular torticollis (fibromatosis colli), a follow-up. Front Neurol. (2016) 7:7. 10.3389/fneur.2016.00007 26869987 PMC4738269

[10] Hong SK Song JW Woo SB Kim JM Kim TE Lee ZI. Clinical usefulness of sonoelastography in infants with congenital muscular torticollis. Ann Rehabil Med. (2016) 40(1):28–33. 10.5535/arm.2016.40.1.28 26949666 PMC4775755

[11] Plominski J Olesinska J Kamelska-Sadowska AM Nowakowski JJ Zaborowska-Sapeta K. Congenital muscular torticollis-current understanding and perinatal risk factors: a retrospective analysis. Healthcare (Basel). (2023) 12(1):13. 10.3390/healthcare12010013 38200919 PMC10778664

[12] Wang L Zhang L Tang Y Qiu L. The value of high-frequency and color doppler ultrasonography in diagnosing congenital muscular torticollis. BMC Musculoskelet Disord. (2012) 13:209. 10.1186/1471-2474-13-209 23102117 PMC3487867

[13] Hwang J Khil EK Jung SJ Choi JA. Correlations between the clinical and ultrasonographic parameters of congenital muscular torticollis without a sternocleidomastoid mass. Korean J Radiol. (2020) 21(12):1374–82. 10.3348/kjr.2019.0893 32729272 PMC7689138

[14] Ge Y He Z Zhang X Chen X. Application of ultrasonography in diagnosis and treatment of children with congenital muscular torticollis. J Family Med Prim Care. (2024) 13(4):1165–8. 10.4103/jfmpc.jfmpc_633_23 38827722 PMC11141958

[15] Chon SC Yoon SI You JH. Use of the novel myokinetic stretching technique to ameliorate fibrotic mass in congenital muscular torticollis: an experimenter-blinded study with 1-year follow-up. J Back Musculoskelet Rehabil. (2010) 23(2):63–8. 10.3233/BMR-2010-0251 20555118

[16] Zorlular R Burak SE Beyoglu R Elbasan B. Motor development and sensory processing skills in infants with congenital muscular torticollis: a cross-sectional study. Eur J Pediatr. (2025) 184(10):630. 10.1007/s00431-025-06457-2 40986093

[17] Kim OH Lee SW Ha EK Kim JH Jo YH Rhie S. Neurodevelopmental outcomes and comorbidities of children with congenital muscular torticollis: evaluation using the national health screening program for infants and children database. Clin Exp Pediatr. (2022) 65(6):312–9. 10.3345/cep.2021.01417 34886594 PMC9171465

[18] Ohman A Nilsson S Beckung E. Stretching treatment for infants with congenital muscular torticollis: physiotherapist or parents? A randomized pilot study. PM R. (2010) 2(12):1073–9. 10.1016/j.pmrj.2010.08.008 21145518

[19] He L Yan X Li J Guan B Ma L Chen Y. Comparison of 2 dosages of stretching treatment in infants with congenital muscular torticollis: a randomized trial. Am J Phys Med Rehabil. (2017) 96(5):333–40. 10.1097/PHM.0000000000000623 27820728

[20] Kim E Choi J Min SY. Combined effect of external treatment of herbal medicine and tuina in congenital muscular torticollis: systematic review and meta-analysis. Medicine. (2022) 101(48):e32086. 10.1097/MD.0000000000032086 36482534 PMC9726427

